# Detection of Antibiotics and Evaluation of Antibacterial Activity with Screen-Printed Electrodes

**DOI:** 10.3390/s18030901

**Published:** 2018-03-18

**Authors:** Florentina-Daniela Munteanu, Ana Maria Titoiu, Jean-Louis Marty, Alina Vasilescu

**Affiliations:** 1 Faculty of Food Engineering, Tourism and Environmental Protection, “Aurel Vlaicu” University of Arad, Elena Dragoi, No. 2, Arad 310330, Romania; florentina.munteanu@uav.ro; 2 International Centre of Biodynamics, 1B Intrarea Portocalelor, Bucharest 060101, Romania; tanamaria@biodyn.ro (A.M.T.); avasilescu@biodyn.ro (A.V.); 3 BAE Laboratory, Université de Perpignan via Domitia, 52 Avenue Paul Alduy, 66860 Perpignan, France

**Keywords:** antibiotic, bacteria, antibiotic susceptibility, screen-printed electrodes

## Abstract

This review provides a brief overview of the fabrication and properties of screen-printed electrodes and details the different opportunities to apply them for the detection of antibiotics, detection of bacteria and antibiotic susceptibility. Among the alternative approaches to costly chromatographic or ELISA methods for antibiotics detection and to lengthy culture methods for bacteria detection, electrochemical biosensors based on screen-printed electrodes present some distinctive advantages. Chemical and (bio)sensors for the detection of antibiotics and assays coupling detection with screen-printed electrodes with immunomagnetic separation are described. With regards to detection of bacteria, the emphasis is placed on applications targeting viable bacterial cells. While the electrochemical sensors and biosensors face many challenges before replacing standard analysis methods, the potential of screen-printed electrodes is increasingly exploited and more applications are anticipated to advance towards commercial analytical tools.

## 1. Introduction

Pharmaceuticals are increasingly used worldwide to ensure public health, a reason for which a large number of active compounds is used to prevent/treat human and animal diseases [1], but their development implies implementation of a rigorous waste management system [2]. 

A considerable increase has been recorded in the use of antibiotics, which is generally due to their specific activity against bacteria or fungi in human and animal hosts and, additionally, due to their ability to increase growth rates and improve feed efficiency [3] in the field of animal husbandry. The group of antibiotics includes natural molecules produced by bacteria and fungi (e.g., benzylpenicillin and gentamicin), semi-synthetic ones, i.e., chemically modified natural antibiotics to increase their stability or synthetic products. Depending on their chemical structure, antibiotics can be classified into several groups: beta-lactams (e.g., penicillin), macrolides (erythromycin), tetracyclines, quinolones (ciprofloxacin), aminoglycosides (e.g., kanamycin), sulfonamides (e.g., sulfadiazine) glycopeptides and oxazolidinones [4]. 

Their presence in the environment is contributing to the increase in the number of multi-resistant bacteria, with subsequent serious implications for human and animal health [5]. Consequently, many countries have imposed maximum residue limits (MRLs) for antibiotics in foodstuffs of animal origin, which vary in the ppb–ppm range, depending on the molecule and the food matrix. Some antibiotics are banned for animal use in some parts of the world while still allowed in others—as in the case of chloramphenicol in the EU or enrofloxacin in the US.

In this context, sensitive analytical methods are required, not only for the quantitative determination of antibiotics in food and the environment, but also for the detection of bacteria, screening compounds with antibacterial activity and for antibiotic susceptibility testing. The most used methods for the detection of antibiotics are chromatographic [6,7,8,9], due to the automation, accurate quantification, simultaneous detection, and the high specificity based on the structural information of the analytes. Other methods for antibiotic determination include electrophoresis [10,11,12,13,14], diode array [15,16] or enzyme immunosorbent assay (ELISA) [17,18,19]. Meanwhile, for the detection of bacteria, the culture-based method remains the golden standard, although a good number of methods based on nucleic acid (e.g., based on polymerase chain reaction (PCR)) or immunologic reactions have been developed. 

Despite the performances of the well-established standard methods for antibiotics and for bacteria detection, these methods are suffering of some well-known disadvantages related to costly equipment and consumables, as well as laborious work that includes the sample preparation and the need for a well-trained technical staff [20].

In this context, electrochemical methods have the advantages of allowing a reliable, fast and portable, in-field analysis to detect a growing range of environmental pollutants, including antibiotics [21,22,23,24,25]. A special place is occupied by biosensors, which are analytical devices that have the advantage of having high selectivity and rapid detection [26,27]. Biosensors achieve selective detection of analytes in complex mixtures without resorting to separation methods, by combining a specific biorecognition element (antibody, peptide, protein, cell, aptamer etc.) with a sensitive physical transducer. Research in the field of biosensors for the detection of antibiotic residues and of bacteria has intensified in the last years, mainly due to the use of new nanomaterials for the construction of these biodevices [28,29] and the selection of novel biorecognition elements with increased selectivity and affinity, e.g., aptamers [20].

Among various types of electrodes used as transducers in electrochemical biosensors, screen-printed electrodes (SPE) [30,31] have attracted special attention because they allow sensitive and selective analysis at a low cost that affords their use as disposable devices, are appropriate for the analysis of low sample volumes, can be integrated in different fluidic setups (e.g., in flow injection analysis) and are conveniently used in combination with magnetic-based separation [32]. The commercial success of glucose biosensors stands as a solid confirmation of the feasibility of electrochemical devices based on SPEs. In addition, SPEs have the advantage that they can be mass-produced easily, without any preliminary laborious preparation steps or requirement for highly qualified personnel. SPEs are versatile devices that can be used in conjunction with simple, portable electrochemical equipment and with various electroanalytical methods such as amperometry [33,34,35,36], voltammetry (differential pulse voltammetry (DPV), square wave voltammetry (SWV), cyclic voltammetry (CV) and linear sweep voltammetry (LSV) [37,38,39,40,41,42,43,44,45,46,47]), potentiometry [48] or EIS [49,50]. This review addresses recent advances made available by using screen-printed electrodes for detection of antibiotics as well as for the evaluation of antibacterial activity. The assessment of antibacterial activity of different compounds typically relies on culture-growing tests and colony counting to estimate the proportion of viable bacteria. However, alternative biosensor-based approaches have also been described for the detection of viable bacteria and screen-printed electrodes have been employed with some of the sensing concepts. Antibiotic susceptibility testing is another area where screen-printed electrodes have been successfully employed and is also discussed below. 

## 2. Screen-Printed Electrodes

Recent years have witnessed the emergence of a wide variety of screen-printed electrodes modified with different materials and printed in various configurations. The current needs for reproducibility, linear range, sensitivity and selectivity require fabrication of chemical sensors and biosensors that are simple to be produced, with low power consumption, cost-effectiveness for the analysis, and versatile for different analytes. Moreover, there are several situations (e.g., on-spot, in situ monitoring, etc.) when the sensors should be directly used without any pre-treatment or cleaning between measurements. 

Screen-printing technology allows the fabrication of a wide range of screen-printed electrodes that are nowadays commercially available [51]. Moreover, this technology enables immobilization of biomolecules onto the electrode surface for selective and disposable biosensors [52]. Compared to other types of electrodes, SPEs have the advantage that many electrodes can be modified simultaneously in a reproducible manner using inks that include nanomaterials, mediators, etc., to achieve high sensitivity in biosensing. While other simple electrodes are investigated for fabrication of flexible, low cost electrochemical devices, e.g., hand-drawn pencil electrodes [53], SPEs have the advantage of batch-to batch reproducibility [53]

**Figure 1:**
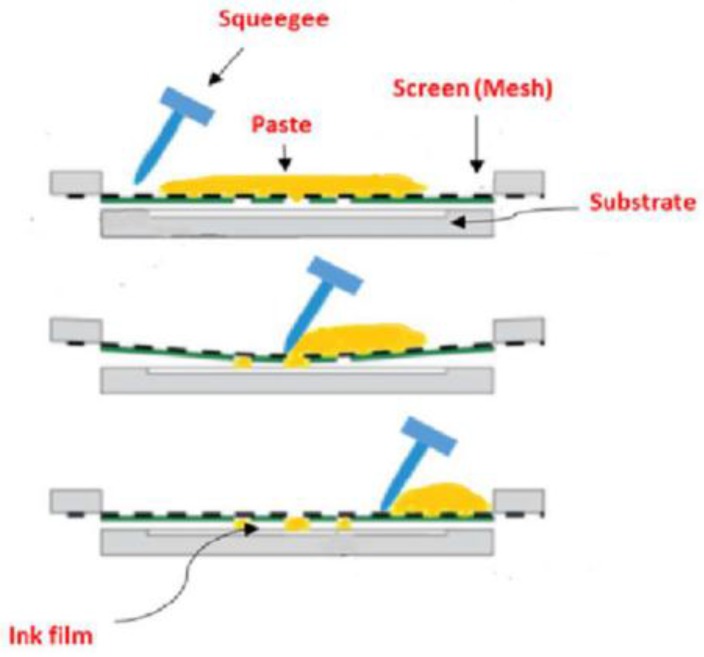
Schematic diagram of the basic screen-printing process for electrodes manufacturing. Reprinted from [54], with permission from Elsevier.

SPEs fabrication consists in the deposition of thin layers of ink on a solid substrate by forcing the ink through a mesh (Figure 1). Layers of ink of different materials can be deposited consecutively using screens of customized design and many electrodes are produced at the same time. This mode of preparation allows an automated process for the production of flexible designs with a very good reproducibility [54].

**Figure 2:**
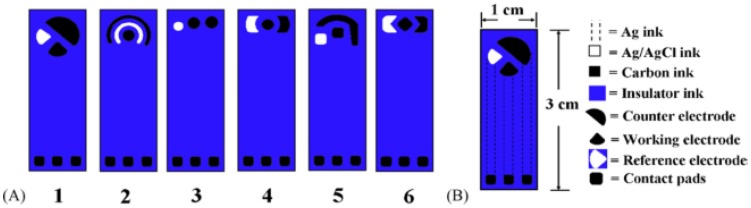
(**A**) Screen-printed sensor chip with six configurations; and (**B**) structure details for configuration 1. Reprinted from [60], with permission from Elsevier.

Various electrode geometries and microelectrode arrays can be realized by screen printing by simply adapting the screen design. The typical format of screen-printed electrochemical devices corresponds to a classic three-electrode cell, comprising working, counter and reference electrodes printed on the same substrate in different configurations, as illustrated in Figure 2 [55,56,57,58,59,60]. 

Two-electrode systems are used in potentiometric sensors or in some amperometric ones where the reference electrode also plays the role of counter electrode. However, with respect to detection by voltammetry sensors, three-electrode systems are required; moreover, they have inherent enhanced sensitivity compared to two-electrode ones [61]. Multi-electrode systems and electrode arrays are currently available commercially. For a more extensive discussion of designs and materials of SPEs, as well as their applications in electrochemical biosensors, the reader is referred to several reviews [32,54,56,62,63,64].

Traditionally, the reference electrode consists of a Ag or Ag/AgCl layer [65], while the counter electrode is usually C or Au. With regards to fabrication of working electrodes, the most used ink and the least expensive is carbon, presenting low background current, wide potential window, chemical inertness, and multiple functionalization possibilities [56,66]. Furthermore, carbon-based electrodes can be easily modified with different nanomaterials or mediators by mixing these in the printing inks. Different carbon based SPEs were fabricated for determination of natural and synthetic antibiotic using: multi-walled carbon nanotubes for determination of sulfonamides [35] and gentamicin sulfate [48]; carbon for simultaneous detection of tetracyclines and sulfonamides [34], penicillins [36,49] or gemifloxacin [39; graphene for tetracycline [43]; graphene/polyaniline nanocomposite for sulfonamides [35]; or gold nanoparticles (AuNPs) for the detection of sulfamethoxazole [33] and epirubicin [45].

Besides carbon, gold and platinum inks are also employed to print working electrodes, being attractive, despite the higher cost, for their catalytic properties, high conductivity, high mechanical strength [55,67] and offering some unique functionalization opportunities (e.g., chemisorption of thiols on Au, widely employed in biosensing). SPEs for detection of antibiotics that are based on working electrodes made of noble metals include pre-treated platinum screen-printed electrodes (SPEs) for the quantification of the strength of garlic, a natural antibiotic [68], and gold SPEs for the detection of fluoroquinolones [40], streptomycin [41], tetracyclines [42] and cefixime [47].

One aspect of the versatility of SPEs is their ease of modification with enzymes, polymers, metals, complexing agents, mediators, nanomaterials, etc., either by incorporating them in the printing ink or by modifying the printed electrodes. Among the various modifiers, nanomaterials are most frequently considered when developing new ink compositions, as nanomaterials are positively affecting the sensitivity, selectivity, and stability of the electrodes [50], enhancing the number of catalytic sites and the active surface area, conductivity [69,70] as well as the loading capacity with biomolecules. For this reason, a special attention was paid to the use of materials such as carbon nanotubes [48,71], metallic nanoparticles [72,73], nanostructured conductive polymers [56,74], nanocomposites [35,52,75] or semiconductor quantum dots [76].

## 3. Detection of Antibiotics with Screen-Printed Electrodes

Screen-printed electrodes were used in numerous applications for the quantitative determination of antibiotics (Table 1). Direct electrochemical detection of antibiotics was achieved with conveniently modified electrodes (e.g., with nanomaterials) for a better sensitivity of the assay [77] compared to bare electrodes.

Alternatively, to achieve selectivity of the assay, the electrodes were modified with chemical receptors such as calixarenes [48] or with biorecognition elements such as penicillin-binding protein [36] or with aptamers [41,46,78]. Aptamers, short oligonucleotides selected in vitro to bind with high affinity and specificity a target analyte, are increasingly researched for antibiotic detection. Aptamers have better stability than antibodies and their production is more reproducible and involves lower cost than for antibodies, which recommend them as replacements for antibodies in commercially available kits and devices. The electrochemical methods used to determine antibiotics in food and biological samples (Table 1) included amperometry [33,34,35,36], various voltammetry techniques (DPV, LSV, and SWV) [37,38,39,40,41,42,43,44,45,46,47], potentiometry [48], electrochemical impedance spectroscopy [49,50] and electrochemiluminescence [76,79]. 

The research results summarized above reflect the utility of screen-printed electrodes for the detection of antibiotics in different sensing configurations, starting from the direct electrochemical oxidation of antibiotics [37] to complex biosensors incorporating complicated arch-shaped or M-shaped DNA constructs [41,42], with emphasis on novel electrode materials and nanomaterial modifiers to improve selectivity and sensitivity. Another simple approach where screen-printed electrodes keep a good advantage over other electrodes is the coupling of electrochemical detection with immunomagnetic separation. Finally, various aptamer-based sensing concepts were developed with different complexity and, given the current trend towards developing new aptamers for antibiotics and the recent emergence of aptamer-based testing kits in the food analysis field (e.g., for mycotoxins, available from NeoVentures Biotechnology Inc., London, ON, Canada), the future will bring novel analytical tools for antibiotics where antibodies will be replaced by aptamers. In the development of such biosensors, the possibilities for functionalizing the screen-printed electrodes in a manner compatible with mass production will play a central role.

### 3.1. Detection of Antibiotics by Amperometry

The amperometric method is performed at a constant potential applied to the working electrode, and, subsequently, the current generated by the oxidation/reduction of the electroactive species is measured. 

One of the developed amperometric sensors for determination of sulfamethoxazole is based on the cross-linking of tyrosinase on screen-printed carbon electrodes that were previously modified with gold nanoparticles [33]. The obtained biosensor was used at an applied potential of +500 mV vs. Ag/AgCl and can be also used for the detection of this antibiotic from water samples. The detection limit for sulfamethoxazole was determined to be 22.6 ± 2.1 µM [33]. 

Conzuelo et al. designed an amperometric immunosensor obtained by covalent binding of Protein G on the SPE surface through a peptide bond, after grafting a film of 4-aminobenzoic acid (4-ABA) onto the carbon working electrode surface. The obtained immunosensor was used for the determination of tetracycline and sulfapyridine [34]. The amperometric signal was measured at −200 mV vs. a silver pseudo-reference electrode, and the detection limits for both antibiotics were determined to be in low ppb levels in untreated milk samples. 

Another application of the screen-printed electrodes was the coupling of the electrochemical detection with a chromatographic separation step to ensure selectivity. For example, this was demonstrated using an in-house working carbon electrode modified with graphene/polyaniline by electrospraying [35]. This experiment was performed for detection of eight sulfonamides in shrimps. The amperometric response was measured at an applied potential of +1.4 V, a potential that was experimentally determined from hydrodynamic voltammetry. The detection limits for the analyzed sulfonamides were in the range 1.162–6.127 ng/mL. It has to be pointed out that prior the amperometric detection, the shrimp samples were cleaned-up through solid-phase extraction and preconcentrated by UPLC separation [35].

Gamella et al. [36] proposed an amperometric method for the detection of residues of β-lactams from milk using an affinity disposable magnetosensor, based on recombinant penicillin-binding protein immobilized on magnetic beads that were kept at the surface of a SPEs [36] with a magnet. The sensor displayed a broad selectivity to β-lactam antibiotics but was insensitive to other antibiotics that may be found in milk such as sulfapyridine, enrofloxacin and tetracycline. The assay principle relied on the competition between free penicillin G from the sample and penicillin G marked with horseradish peroxidase (HRP), which competed for binding to the PBP on the magnetic beads at the electrode surface. The amount of penicillin in the sample was correlated with the activity of the labeled HRP, measured via the enzymatic substrate H_2_O_2_, and the electrochemical mediator hydroquinone. The current intensity due to the reduction of hydroquinone was thus correlated with the amount of antibiotic in the samples. The obtained detection limits for six antibiotics were in low ppb levels in the case of untreated milk samples.

### 3.2. Voltammetry Detection of Antibiotics

In voltammetry, the potential of the working electrode is varied across a specified range and the current intensity is measured to obtain information about the nature and amount of the electroactive species present in the electrochemical cell, either in the solution or at the electrode surface. The different techniques—CV, LSV, SWV, and DPV—differ in the manner of varying the potential; number of scans performed, i.e., single or repetitive; and the way the intensity of electrical current is measured. 

**Figure 3:**
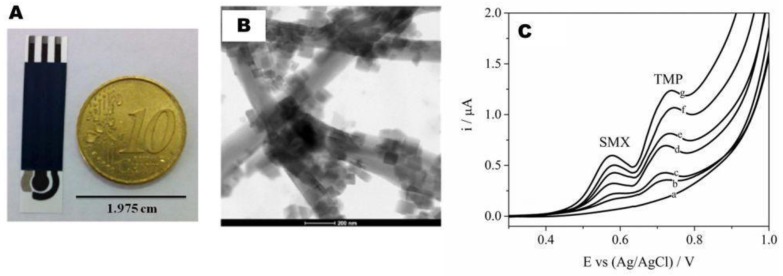
(**A**) Image of the screen-printed sensor including a carbon working electrode modified with MWCNT-PBNC (in the center), a Ag/AgCl reference (left) and a carbon counter electrode (right side). (**B**) Transmission electron microscopy (TEM) image of MWCNT-PBNC composite used for working electrode modification. (**C**) DPV of MWCNT-PBNC-SPE in 0.04 mol L^−1^ Britton Robinson buffer pH 7.0 in equimolar mixtures of SMX and TMP with increasing concentrations: (a) 0 µmol L^−1^; (b) 1 µmol L^−1^; (c) 2 µmol L^−1^; (d) 4 µmol L^−1^; (e) 6 µmol L^−1^; (f) 8 µmol L^−1^; and (g) 10 µmol L^−1^. Adapted from [37] with permission from Elsevier.

In a simple approach amenable to the detection of electroactive antibiotics, electrochemical sensors have been developed based on SPEs without using any biological materials and relying exclusively on the intrinsic electroactivity of the antibiotics themselves. Such an example is the sensor modified with multi-walled carbon nanotubes decorated with Prussian blue nanocubes (MWCNT-PBNC) [37] illustrated in Figure 3A, featuring, besides the MWCNT-PBNC working electrode, a carbon counter electrode and a silver/silver chloride reference electrode. The fabrication of the working electrode included the manual drop-casting of the MWCNT-PBNC composite on the screen-printed carbon electrode as a final step. The nanocomposite consists in MWCNTs uniformly covered by 50 nm PB nanocubes (Figure 3B). Compared to bare carbon electrodes, the MWCNT-PBNC-modified ones afforded a negative shift of the oxidation peak potential for sulfamethoxazole (SMX) and trimethoprim (TMP) of three and two times higher current intensities, respectively. The electrocatalytic effect was due to a combination between the high surface area of MWCNT and the presence of numerous catalytic sites on the edges of PBNC that facilitated the formation of Fe(III) complexes with SMX and TMP. The sensor allowed selective detection of SMX and TMP in binary mixtures and spiked synthetic urine, with detection limits of 36 nM and 60 nM, respectively. The method is relevant for analysis of such antibiotic combinations that are used to boost the treatment efficacy and which are known beforehand. The oxidation potentials of SMX and TMP are separated enough to afford such analysis (Figure 3C).

However, for unknown samples, there are many compounds oxidizing in the same potential range that could potentially interfere in the assay and this is the general limitation of electrochemical sensors lacking recognition elements. A similar strategy was used for the quantitative determination of gemifloxacin in pharmaceutical tablets by DPV, by direct electrochemical oxidation on a screen-printed carbon electrode [39]. The pharmaceutical excipients have not interfered in the assay. Nonetheless, for other types of samples (food and biological) with complex matrices, appropriate sample pre-treatment or separations will have to be devised to avoid interfering compounds that affect the accuracy of the measurements. A detection limit of 0.15 µM for gemifloxacin was reported. 

The necessity to determine the presence of antibiotics in some edible tissues lead to the development of a competitive ELISA assay for the detection of tylosin and erythromycin from bovine muscle with electrochemical detection by DPV on screen-printed electrodes [38]. In this experiment, the antibodies for tylosin and erythromycin were labeled with the enzyme alkaline phosphatase (ALP). The quantitative detection of antibiotics is ultimately related to the oxidation current of p-aminophenol (PAP), resulting in the conversion of p-aminophenol phosphate, catalyzed by ALP. The determined detection limits for erythromycin and tylosin were 0.2 ng/mL and 1 ng/mL, respectively. 

The selective detection of ciprofloxacin in foodstuff and environmental samples [40] was achieved with a competitive assay based on an aptasensor. This aptasensor was based on an aptamer as molecular recognition probe immobilized on a gold electrode and a single-stranded DNA-binding protein that binds to the aptamer, thus forming a physical and negatively charged barrier for the access of redox probe ([Fe(CN)_6_)]^3−/4−^) to the surface of the gold electrode. In the presence of ciprofloxacin, the aptamer binds to its target, which, consequently, is no longer available for interaction with the single-stranded DNA-binding protein and the current recorded via cyclic voltammetry of ferricyanide is higher. The developed sensor was successfully used for the detection of ciprofloxacin by differential pulse voltammetry from spiked milk, serum, and water, with detection limits of 351, 336 and 261 pM, respectively [40]. 

Filik et al. [43] are reporting a reliable, modest cost, portable, easy to operate and small system that is based on graphene modified screen-printed carbon paste electrode. The sensor can be used for the voltammetric detection of tetracycline from milk and honey samples. The detection limit was found to be 0.08 µM, and the tested milk and honey samples did not contain tetracycline. The recoveries of the antibiotic from spiked samples were between 98% and 102%, confirming the accuracy of the sensor.

Another aptasensor for detection of tetracycline was obtained through the immobilization of biotinylated ssDNA aptamer on a streptavidin-modified screen-printed gold electrode [46]. The sensor is characterized by a low detection limit (10 nM) and the possibility to be used for determination of tetracycline in drinking water, food products, and pharmaceutical preparations.

Zhan et al. [44] described a screen-printed carbon electrode modified with a composite consisting of reduced graphene oxide, magnetite, and sodium alginate onto which was bound the tetracycline aptamer. Detection of tetracycline through differential pulse voltammetry led to a very good detection limit of 600 pM. The developed sensor was proved to be an appropriate platform for the analysis of tetracycline from real samples. 

A screen-printed gold electrode modified with cysteine self-assembled monolayers on gold nanoparticles was used for rapid and simultaneous determination of tetracycline and cefixime from milk, honey and serum by means of square wave voltammetry [47]. This sensor combined with chemometric tools is a good candidate for detection of the targeted antibiotics from biological fluids. The detection limits for tetracycline were found to be between 0.42 and 0.52 µM, while for cefixime the limits are between 0.32 and 0.38 µM.

**Figure 4:**
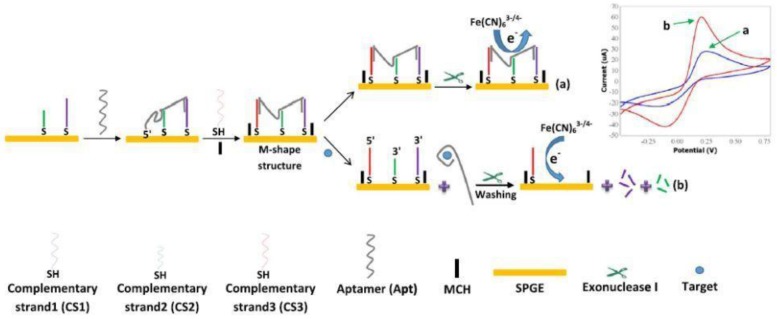
Schematic illustration of tetracycline detection based on electrochemical method. In the absence of tetracycline, the M-shape structure of Apt-CSs complex remains intact and redox probe could not have access to the surface of electrode, leading to a weak electrochemical signal (**a**). In the presence of tetracycline, Apt binds to tetracycline and leaves the CSs. Exo I degrades CS1 and CS2, resulting in the free access of redox mediator to the surface of electrode and a strong electrochemical signal (**b**). Reprinted from [42] with permission from Elsevier.

Gold screen-printed electrodes were modified with a M-shaped DNA construct made from a tetracycline (TCN) aptamer partially hybridized with three complementary strands (CS). The binding event with tetracycline released the aptamer from the DN construct. Moreover, the enzyme exonuclease I was added to the medium to degrade the complementary sequences CS1 and CS2 remaining on electrode surface, thus facilitating the access of the redox probe, ferricyanide, to the electrode surface. The net effect is an increased electrochemical signal, proportional with the amount of tetracycline in the sample [42] (Figure 4). 

This sensor design enabled maximizing the differences in the current intensity of ferricyanide in the absence and the presence of the target antibiotic. The aptasensor was applied for detection of TCN from serum and milk, and the measurement accuracy for samples that contain proteins and other interfering materials was demonstrated by recoveries of 93.1% and 103.8% from spiked samples. Despite the complexity of these matrices containing proteins and other interfering materials, the sensitivity of the sensor was similar to the sensitivity obtained for determinations in the buffer; only the detection limits in milk and serum were somewhat higher, around 0.7 nM, compared to 0.45 nM in the buffer [42]. 

An electrochemical aptasensor for the detection of streptomycin [41] was based on an equally interesting DNA construct. Thiolated streptomycin aptamer and a complementary strand were immobilized on a gold electrode and partially hybridized in such a manner to form together an arch-like structure that acts as a barrier to electron transfer from the redox species ferricyanide, added in solution. When streptomycin is added the aptamer is displaced from this structure to bind its target. Furthermore, as the arch-like structure is destroyed, the addition of exonuclease I to degrade the cDNA remaining on the electrode surface further enables the diffusion and electron transfer of ferricyanide at the electrode surface. As in the previous example, an enhanced electrochemical signal is observed, proportional to the concentration of the antibiotic. The concept was verified for the detection of streptomycin from milk and blood serum through differential pulse voltammetry, with detection limits of 14.1 and 15.3 nM, respectively [41].

### 3.3. Potentiometric Detection 

Potentiometric detection is a simple method that presumes measuring the differences in voltage between an indicator electrode, whose potential is correlated with the concentration of ions in solutions and a reference electrode, typically in the absence of electrical current flow in the electrochemical cell. To ensure selectivity, the indicator electrode is modified with a ionophore, selective for the ion of interest.

Khaled et al. developed a simple, selective and sensitive disposable potentiometric sensor for detection of gentamycin sulfate, by modifying SPCEs with multi-walled carbon nanotubes–polyvinyl chloride in presence of calix [4] arene as ionophore [48]. Calixarene is the key constituent of the sensor to achieve selectivity, as it forms an inclusion complex with the gentamicin cation. The detection limit for gentamicin-sulfate with this potentiometric SPE was found to be 7.5 × 10^−8^ mol/L. A notable feature of the developed method resides in the fact that the average recovery of gentamycin from spiked water samples is comparable to values reported with the standard methods [48].

### 3.4. Detection by Electrochemical Impedance Spectroscopy

Electrochemical impedance spectroscopy (EIS) [80] is a label-free, sensitive method, widely used to assess the changes in the electrical properties at the sensor–sample interface. In faradaic EIS, a redox probe, typically [Fe(CN)_6_]^3−^/^4−^, is added in the solution and, over a fixed potential value, corresponding to the formal potential of [Fe(CN)_6_]^3−^/^4−^, is superposed a small ac signal of small amplitude (5–10 mV). The complex impedance of the system, defined as the ratio between voltage and current is measured at different frequencies. The electrode/solution system is assimilated with an equivalent electrical circuit including resistive, capacitive and inductive components. Appropriately fitting the impedance data to this electrical circuit allows calculating the resistance to charge transfer R_CT_ at the electrode interface, among others. R_CT_ is the most used parameter to describe quantitatively the changes in electrical properties following the antibiotic binding to the sensor as this event affects the flow of electrons to the electrode surface. 

By using this strategy, the label-free detection of penicillin G from milk was achieved with selective DNA aptamers [49]. To ensure accurate results, the electrodes were protected against non-specific adsorption by coating with 2% casein and the milk samples were treated with isopropanol and filtered through a 0.45 µm membrane. Recoveries of 83–100% were reported from milk samples spiked with 2–10 ppb penicillin G. The linear range was 0.4–1000 ppb, appropriate for analysis in milk where the maximum MRL is currently set at 4 ppb.

Another label-free aptasensor was constructed for the detection of epirubicin by self-assembling a thiolated aptamer on carbon screen-printed electrode modified with electrodeposited gold nanoparticles on magnetic double-charged diazoniabicyclo [2.2.2] octane dichloride silica hybrid [45]. The aptasensor is characterized by a detection limit of 40 nM and its applicability for the detection of epirubicin from human blood serum was successfully proven.

Sharma et al. [50] reported the first impedimetric sensor designed for the detection of kanamycin. This aptasensor is based on a screen-printed carbon electrode functionalized with an in vitro selected single strand DNA (ssDNA) anti-kanamycin-aptamer. The sensor has the advantages of being label-free and portable [50]. The dynamic range of the sensor is 1.2–600 ng/mL, while the detection limit for kanamycin is 0.11 ng/mL, complies with the regulatory standards for this antibiotic (MRL limit is 150 ng/mL). 

Detection of tetracycline was also performed by using two different aptasensors that were based on modified carbon paste/oleic acid and magnetic bar carbon paste/Fe_3_O_4_@oleic acid nanoparticle. After the modification of the electrodes with aptamers, a detection limit of 3.0 × 10^−13^ M was obtained for tetracycline determination through the electrochemical spectroscopy impedance for both used aptasensors [50].

### 3.5. Electrochemiluminescent Detection

Electrochemiluminescence (ECL) is a very sensitive method, where chemiluminescence is generated by application of an electrical potential in a medium containing luminescent species (e.g., quantum dots). ECL combines the sensitivity of chemiluminescent detection, due to the lack of background luminescent signal, with the possibility to modulate the selectivity of detection by controlling the applied potential.

Feng et al. [76,79] developed electrochemiluminescent aptasensors for the detection of chloramphenicol. The “dual-potential” electrochemiluminescence aptasensor array had two working electrodes, and their surface was modified with CdS quantum dots and luminol-gold nanoparticles to obtain the cathode and anode electrochemiluminescent emitters, respectively [79]. Besides the fact that this aptasensor has the advantage of not giving a false positive signal, it can also be used for the simultaneous detection of chloramphenicol and malachite green, with a detection limit of 0.03 nM for chloramphenicol [76,79] and 0.07 nM for malachite green [76].

## 4. Evaluation of Antibacterial Activity with Methods Using Screen-Printed Electrodes 

The challenges brought forth by outbreaks due to foodborne pathogens, the prevalence of nosocomial infections and the increase in resistance to antibiotics worldwide focused a significant part of research efforts into finding fast ways for: (i) detecting bacteria with high sensitivity; (ii) screening the antibacterial activity of various compounds; and (iii) evaluating the sensitivity of bacteria to antibiotics, i.e., antibiotic susceptibility testing (AST). Evaluation of the antibacterial activity presumes a method that is not only specific to that particular bacterial strain but also able to differentiate between live and dead bacterial cells. Differentiation can be made based on: growth, metabolism, membrane permeability, etc., thus it is traditionally achieved by plate-counting methods and more recently by multi parameter flow cytometry where several fluorescent dyes may be included to assess cell viability, structural integrity of the cell membrane, and membrane potential at a single-cell level [81]. Emerging methods for antibiotics susceptibility tests and the commercially available tests were summarized in recent reviews [82,83].

To shorten the analysis time for the detection of bacteria various methods based on nucleic acids, immunological assays and biosensors have been developed [84,85,86]. While DNA and immunoassays are sensitive, selective and faster than classic culture-based methods, they presume costly instrumentation, significant cost per assay, possibly suffer from interferences from real samples, and/or cannot differentiate live/dead bacteria, all of which prevent their application on a wider scale. There was a significant progress in development of microfluidic devices with complex capabilities for bacteria isolation, cell lysis and DNA amplification for identifying live bacteria [87]. Polymerase chain reaction (PCR), [88] dielectrophoresis [89,90], and Surface Enhanced Raman Spectroscopy (SERS) [91] are among the other modern techniques that have been integrated with microchips or biosensors. Moreover, different forms of electrical evaluation of cell viability have been proposed, e.g., monitoring the variation of electrical conductance of evaporating droplets of bacteria enabling to probe the osmo-regulatory mechanism functioning in living cells [92].

In this context, electrochemical biosensors have been advanced as modern, versatile analytical tools answering the need for fast, simple, cost-effective and specific detection of bacteria, with the added advantage of the possibility of integration in sensor arrays and in complex microfluidic setups for high-throughput analysis.

### 4.1. Biosensing Approaches for the Detection of Bacteria and AST

Various biosensors have been developed for the detection of bacteria based on electrochemical, optical, piezoelectric, etc., methods [93,94]. The choice of biorecognition element is critical for the ability to differentiate between live and dead bacteria. As noted by Templier et al. [95], in most cases, the biorecognition elements are developed using as target dead or inactivated bacterial cells. Therefore, discrimination solely based on differences in affinity between live and dead bacteria is not enough and coupling with a culturing step to prove the viability of bacteria in the sample is necessary. Only in a few cases (i.e., with bacteriophage [78,96], antibodies [97] or aptamers [78,98]) it was claimed that the biosensors allowed a direct discrimination between live and dead bacteria. For example, a low-cost platform able to detect live bacteria in less than 10 min combines the specificity of bacteriophages with the sensitivity of integrated potassium-sensitive field-effect transistors (ISFETs) [96]. An impedimetric immunosensor based antibody-modified polysilicon interdigitated electrodes detected specifically viable bacteria with a concentration of 3 × 10^2^ cfu mL^−1^ or higher within 1 h [97]. It is also worth mentioning the Hemag1P aptamer used in an optical biosensor that bound with higher affinity live *L. acidophilus* cells compared to heat-denatured ones [98]. Although the aptamer had not yet been used in an electrochemical biosensor, it was suggested that it targets the membrane S-protein of the bacteria and based on this could be useful for live/dead differentiation. This is one example of biorecognition elements that can be combined with SPEs to obtain electrochemical biosensors for assessment of live bacteria. 

Among the different biorecognition elements, bacteriophages are very attractive in biosensors for the detection of bacteria or bacterial lysis products [84]: they have high affinity, display strain specificity for target bacteria and their production involve lower costs than antibodies. In biosensors, bacteriophages were successfully immobilized either by physical adsorption on bare gold or by covalent immobilization on cysteamine-modified, glutaraldehyde-treated gold, or on carbon-based interfaces [99,100]. 

With regards to AST, some of the recent biosensor-based approaches include optical tracking of bacterial motion at single cell level [83], assessment of the mechanical vibrations from the cell walls of bacteria by quartz crystal microbalance [101] or measuring the frequency changes associated with the growth of live cells captured on a phage-coated magnetoelastic biosensor [102]. Additionally, electrochemical detection methods, in particular EIS and voltammetry, were also used with lab-on-a-chip or microfluidic setups devices, e.g., with antibody functionalized surfaces for specific capture of bacteria coupled with impedance measurements [103] or in rapid (within 1 h) detection of antibiotic susceptible strains of *E. coli* (UPEC) and *K. pneumoniae* by DPV. Bacteria was cultured inside nanowells with the antibiotic and an electrochemically active probe (resazurin) was added, that is reduced by metabolic processes in live bacteria [104]. 

Compared to costlier miniaturized chips (e.g., interdigitated array microelectrodes fabricated by photolithography and wet etching), screen-printed devices offer good analytical performances and reproducibility for the detection of bacterial cells at a low cost, which recommends them for this type of application [105]. The next section of the review is focused on the detection of bacteria and AST with SPEs, which is discussed in detail according to the electrochemical method employed.

### 4.2. Applications of SPE-Based Biosensors 

Biosensors based on SPEs have been proposed as useful tools for the detection of bacteria based on antibodies [105,106], aptamers [78], antimicrobial peptides [107] or bacteriophages [99] as recognition elements. MIP-functionalized SPEs were used to detect flagellar filaments located on the outer surface of bacteria [108], endotoxins generated by bacteria [109] or bacterial DNA, e.g., virulence genes imparting resistance to antibiotics. 

The performances of several relevant biosensors based on screen-printed electrodes that were developed for the detection of bacteria and antibacterial susceptibility testing are presented synthetically in Table 2 and discussed in more detail below. As emphasized in Table 2, the preferred electrochemical detection methods were EIS and voltammetry techniques such as SWV and DPV, while amperometry and other methods (e.g., potentiometry and ion-sensitive field-effect transistors (ISFETs) [96] were more rarely used. 

The role of the screen-printed electrodes ranged from a simple platform for electrochemical detection when the pathogen biorecognition step or the incubation with the antibiotic were performed outside the electrochemical cell, prior to electrochemical analysis to more complex configurations where the capture and detection of target bacteria or virulence gene was performed on the adequately functionalized electrodes. Specific detection of viable bacteria was claimed based on differences in affinity observed with live versus dead bacteria or, more rarely, by confirmation through parallel analysis by standard culture methods and antibiotic susceptibility assay.

Several studies reported the analysis of real samples inoculated with bacteria, however most studies were performed on pure cultures and analysis was done by EIS, amperometry, chronocoulometry or voltammetry.

#### 4.2.1. Impedimetric Detection of Bacteria

An impedimetric biosensor for the detection of viable bacteria was reportedly obtained by using screen-printed carbon electrodes, coated with gold nanoparticles and functionalized with an aptamer selected by a stringent process [78]. To reach the required specificity and affinity for live bacteria, the twelve rounds aptamer selection process was directed against live *S. typhimurium* cells and each round included also a negative selection step against *S. typhimurium* inactivated by heat, as well as towards a mixture of other pathogens (*Salmonella enteritidis*, *Escherichia coli*, *Staphylococcus aureus*, *Pseudomonas aeruginosa*, and *Citrobacter freundii*). Impedimetric detection allowed to reaching a detection limit of 600 cfu mL^−1^ and a good selectivity versus other *Salmonella* strains. 

**Figure 5:**
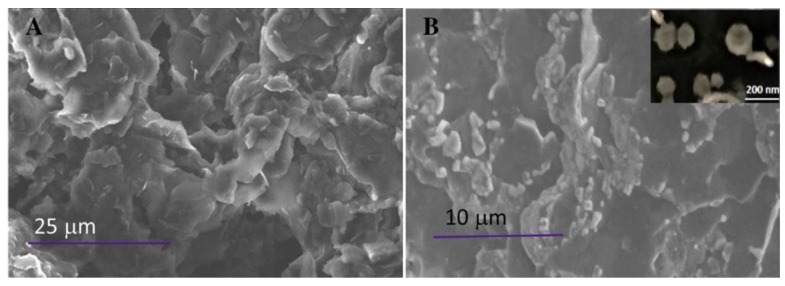
FE-SEM images of: blank SPE (**A**); and bacteriophage immobilized SPE (**B**). Reproduced from [99] with permission from Elsevier.

Bhardwaj et al. [99] described an impedimetric biosensor for *Staphylococcus arlettae* made by covalent immobilization of bacteriophages on a commercial screen-printed graphene electrode. To anchor the bacteriophages, the graphene electrode was oxidized electrochemically 3 min at +1.0 V in 0.1 M HCl to introduce a high number of carboxylic groups, and then the bacteriophage was fixed via carbodiimide chemistry by linking its amine-end group to the carboxylic groups on the electrode. The success of the immobilization procedure was checked by FE-SEM, showing the presence of bacteriophage particles on electrode surface (Figure 5), as well as by FTIR and UV-VIS spectrometry. 

Upon incubation with target bacteria, the bacteria-bacteriophage affinity complex was formed at the electrode surface acting as a diffusion barrier for the ferricyanide redox probe used to measure the electrode impedance. The impedance increased in direct correlation with the magnitude of bacterial concentration. The biosensor allowed fast analysis of *S. arlettae* in only 2 min, with a detection limit of 2 cfu and a range up to 2.0 × 10^6^ cfu. Application for the detection of bacteria in spiked water and apple juice was demonstrated and other advantageous features of the sensor were the good storage stability of three months and the proven selectivity versus strains such as *S. aureus* and *E. coli*. However, the assay was based on the electrochemical monitoring of the binding event between the bacteriophage and *S. arlettae* and not on bacterial lysis, thus the differentiation between live/dead bacteria was not pursued in this study. Nonetheless, the study illustrates the possibility to effectively immobilize bacteriophages on screen-printed graphene electrodes with preservation of their activity and good storage stability. The study did not provide a comparison with bare carbon or other materials to evaluate the advantages of graphene, except for the mention that graphene has a high surface area, high electron mobility and can be enriched with carboxylic groups that served to anchor covalently the bacteriophage. 

In addition to bacteriophages, synthetic engineered antimicrobial peptides have been indicated as specific recognition elements in biosensors for the detection and live/dead differentiation of pathogenic bacteria [119]. For example, this was achieved with a biosensor made by immobilizing the engineered peptides on a gold electrode and monitoring the binding of bacteria to the peptide-modified surfaces by EIS [119]. Such a biosensor design could be easily transferred to screen-printed electrodes.

Not in the least, a label free impedimetric biosensor made by immobilizing bacteria on antibody-functionalized electrodes printed on a plastic microchip was developed for the fast antibiotic susceptibility testing of bacteria [113]. Detection of *Escherichia coli* (*E. coli*) and methicillin-resistant *Staphylococcus aureus* (MRSA) was achieved by non-faradaic EIS and the impedance was monitored in real time during a 1-h incubation of bacteria with the antibiotic. The microchip fabricated by screen-printing and laser cutting has a size of 20 mm × 20 mm (length × width) and includes besides the interdigitated electrodes a heater, a temperature sensor, and microfluidic channels. Thus, the bacteria were isolated on the chip from blood spiked with *E. coli* and MRSA. The concept was proven by following the interaction of *E. coli* and MRSA with six antibiotics with different modes of action, namely ampicillin, ciprofloxacin, erythromycin, daptomycin, gentamicin and methicillin. To validate the approach, parallel analysis by bacteria viability and conventional antibiogram assays were performed.

Another area of application of screen-printed electrodes is in antimicrobial susceptibility testing based on determination of virulence genes, measuring the changes in electrical properties of bacteria incubated with antibiotics or measuring the effect on the metabolic activity of the bacterial cells, i.e., on their respiratory activity. 

Thus, Obaje et al. [57] described a biosensor for the determination of the blaNDM gene encoding for New Delhi metallo (NDM)-beta-lactamase. This gene is present in multi-drug resistant microorganisms, therefore is a primary target in antibiotic susceptibility testing. The biosensor functionalization strategy consisted in the covalent attachment of amine-ended PNA to carboxylic groups electrografted via in situ generated diazonium salt. The biosensor assembly required significantly less time (2 h 40 min) than a similar device based on a gold electrode functionalized with thiolated PNA (17 h 30 min) and had a detection limit of 200 nM bla_NDM_ by EIS. The authors have discussed the influence of graphite and dielectric paste used in screen-printing on sensor performances. 

#### 4.2.2. Detection of Viable Bacteria by Amperometry

Cell viability detection was pursued by detecting enzymes such as β-d-galactosidase, indicators of the bacterial metabolism. In 2003, Neufeld et al. [114] detected viable *Escherichia coli* (K-12, MG1655) by combining phage-typing with amperometric measurements of the activity of β-d-galactosidase released following phage-induced lysis. As the intracellular enzyme was only released by viable bacterial cells at the end of their lifecycle, the method provided proof of the viability of determined bacterial cells. The total duration of the test including culture of bacteria before incubating with the phage was 6–8 h. After incubation with the phage, the suspension of lysed cells was filtered and an aliquot of the solution was analyzed by amperometry using screen-printed carbon electrodes to determine the activity of released β-d-galactosidase. To this end, the enzyme substrate p-aminophenyl-β-d-galactopyranoside was added in the electrochemical cell and the product of the enzymatic reaction, p-aminophenol was detected amperometrically by oxidation at 220 mV on the carbon electrodes. The magnitude of the current recorded by amperometry is directly correlated with β-d-galactosidase activity. An eight-channel multipotentiostat and a system with eight screen-printed electrodes printed on a ceramic support were used in this study. Method selectivity was proven by parallel tests with *K. pneumoniae*, where the intracellular β-d-galactosidase was not released as the bacteria was not lysed by the phage [114]. 

#### 4.2.3. Voltammetry Based Techniques for Viability Testing

**Figure 6:**
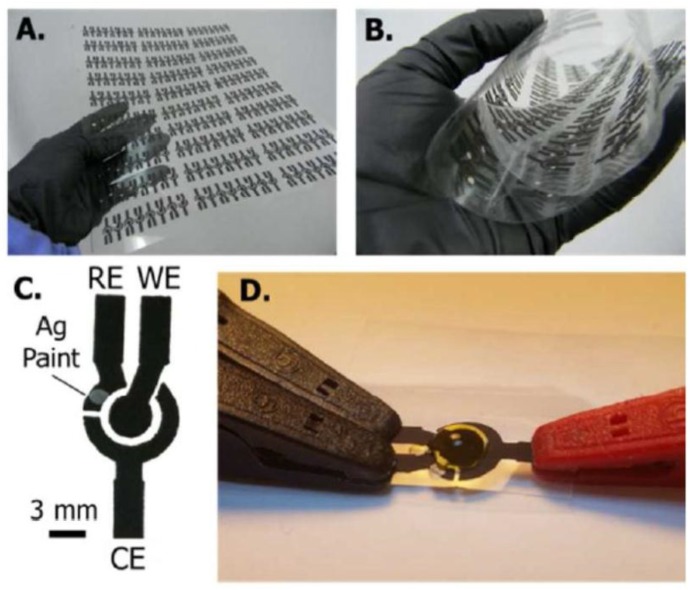
Reprinted from [116] with permission from ACS. Stencil printed carbon electrodes on transparency film shown as a (**A**) printed sheet that is (**B**) flexible. (**C**) A single printed electrode image showing carbon working (WE), silver paint reference (RE), and carbon counter (CE) electrode geometries and connections. (**D**) Final device image with 30 µL of solution contained within the central well and connected to potentiostat leads.

In a similar approach, viable *E. coli, Enterococcus faecalis* and *E. faecium* were detected by measuring the activity of indicator enzymes released from cells lysed by 20 s sonication. Three-electrode electrochemical cells consisting in carbon working and counter electrode and silver reference electrode with the geometry shown in Figure 6A–C were stencil-printed on cheap, flexible transparent film [116] and applied for measuring the activities of β-galactosidase and β-glucuronidase (Figure 5) released from *E. coli* as well as β-glucosidase released from *Enterococcus spp.*

**Figure 7:**
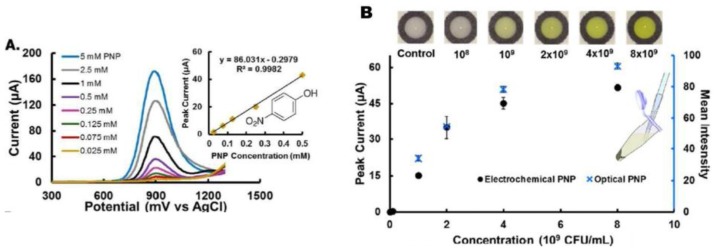
(**A**) SWV for increasing concentration of PAP and calibration plot in PBS buffer pH 6.5; and (**B**) electrochemical and colorimetric response of centrifuged and resuspended *E. faecalis* incubated for 2 h with PNP-gluco substrate. Adapted from [116] with permission from ACS.

A 30 µL aliquot of lysed cells was tested with the electrochemical devices (Figure 5D). The enzyme substrates added in the reaction medium produced p-aminophenol (PAP), o-nitrophenol (ONP) or p-nitrophenol (PNP), who were electrochemically oxidized by SWV. The magnitude of the electrochemical current was linked to the activity of enzymes present in the reaction medium and hence to the concentration of bacteria. In parallel, the authors presented a colorimetric paper-based well plate system using PNP and ONP, developed from a simple cardboard box and smart phone with similar analytical performances (Figure 7). One advantage of the electrochemical method compared to the colorimetric one lies in the possibility to be used with colored samples without being affected by background interference.

The method allowed detecting 10 cfu mL^−1^ of *E. coli* and cfu mL^−1^ of *Enterococcus faecalis* and *E. faecium* in pure cultures after 4 and 8 h pre-enrichment, respectively. Additionally, non-inoculated and inoculated water and alfalfa sprouts were tested. Positive detection of inoculated *E. coli* at 2.3 × 10^2^ cfu mL^−1^ was achieved in 4 h, while 10 cfu mL^−1^ of *E. faecium* was detected after 12-h pre-enrichment. 

**Figure 8:**
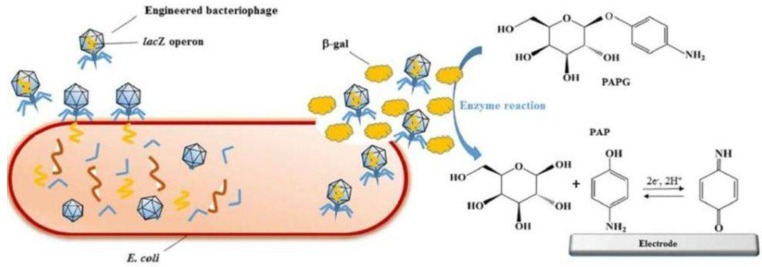
Schematic representation of the electrochemical detection of *E. coli* using engineered phages. Specific capture and infection of *E. coli* by T7lacZ phage resulted in the release and overexpression of β-galactosidase. PAPG was catalyzed by β-galactosidase into PAP which can be quantified with the electrochemical device. Reproduced in part from [115], with permission from ACS.

The current trend is to use engineered phages in combination with cost-effective disposable electrodes to measure the lysis effect (activity of β-galactosidase, linked to the concentration of bacteria in the sample). Engineered phages are used to overexpress β-galactosidase and thus increase the sensitivity of detection (Figure 8). An incubation time of 7 h was necessary to achieve detection of 10^2^ cfu mL^−1^ *E. coli* in aqueous samples (water, skim milk, and apple juice), without any pre-concentration [115]. The activity of β-galactosidase was determined by DPV analysis of the p-aminophenol formed in the β-galactosidase catalyzed conversion of its electrochemically inactive substrate 4-aminophenyl-β-galactopyranoside (PAPG, Figure 8).

#### 4.2.4. Chronocoulometric Detection

To test the antibiotic susceptibility of *Escherichia coli* JM105, Mann et al. [118] measured the cellular respiratory activity with a screen-printed carbon electrode array. The artificial electron mediator ferricyanide has much higher solubility than oxygen and can be used as alternative terminal electron acceptor, enabling sensitive monitoring of bacterial respiration via electrochemistry. Ferricyanide added in the bacterial suspensions was reduced to ferrocyanide due to the bacterial respiration activity and the ferrocyanide formed was detected by oxidation on the screen-printed carbon electrodes polarized at +0.5 V. Further, the decrease in the respiratory activity in the bacterial suspension following a 10 min incubation with an antibiotic was correlated with the amount and type of antibiotic used. A total of 17 antibiotics were screened. To prevent non-specific adsorption of antibiotics, the electrodes were coated with poly-l-lysine or chitosan. While the IC_50_ of 2.0 ± 0.2 mM for chloramphenicol were similar to other respiration-based results for the same microorganism, they were significantly higher than values obtained by growth-based antibiotic susceptibility testing methods [118]. The authors attributed the differences to the very different timescales of the two methods.

### 4.3. Screen-Printed Electrodes Coupled with Magnetic Separation

The current trends in biosensors research include, besides miniaturized setups for high portability and low costs, the use of magnetic manipulation for improving the selectivity and sensitivity in real samples. More specifically, adequately functionalized magnetic beads facilitate extraction and pre-concentration of target bacteria from complex samples but also help improving the signal/noise ratio of the electrochemical detection method, e.g., this can be achieved by periodic actuation of the bacteria-binding beads in magnetic field [120]. In addition to more complex and expensive lab-on-a-chip devices, screen-printed electrodes are also amenable to facile modification with magnetic beads, for the analysis of low sample volumes and integration in fluidic setups. The combination of immunomagnetic separation with detection using screen-printed electrodes leads to improving the detection limit of *E. coli* K12 from 10^4^ to 10^3^ cfu mL^−1^ and facilitated the prevention of non-specific adsorption in complex matrices such as milk [111].

In its 2016 review of magneto immunosensors, Herrasti et al. [121] noted that the advantages of screen-printed devices lie not only in good performances and reproducibility at a low production cost but also in the analysis of small sample volumes, their compatibility with easy modification with magnetic nanoparticles for increased sensitivity and assay simplicity. Detection of *B. cereus* and *E. coli* O157:H7, with detection limits of 40 cfu mL^−1^ and 6 cfu mL^−1^ was reported by coupling screen-printed carbon electrodes with immunomagnetic separation. [122]. Magnetic/polyaniline core/shell nano-particles functionalized with specific antibodies were used to extract the bacteria from the sample and were deposited on the electrode surface and concentrated by placing a magnet under the electrode. Changes in the electrical properties at electrode surface following the deposition of the nanoparticles modified with the immune complexes were readily measured by cyclic voltammetry, the whole test taking little over 1 h [122]. In another example, the combination of immunomagnetic separation with detection with screen-printed electrode has improved the detection limit of *E. coli* K12 from 10^4^ to 10^3^ cfu mL^−1^ and facilitated the prevention of non-specific adsorption in complex matrices such as milk [111]. 

Measurements with screen-printed electrodes were sometimes combined with sample preconcentration with phage-functionalized magnetic beads for increased sensitivity of the assay [111]. Bacteria separated from complex mixtures by binding to functionalized magnetic beads were deposited and concentrated on the electrodes by placing a magnet under the electrode. Detection by impedance and voltammetry is based on the proportionality between the amount of bacteria/lysis products and the changes in the electrical properties at electrode surface, measured via a redox probe such as ferricyanide.

## 5. Conclusions and Perspectives

This review summarizes the recent advances in development and applications of screen-printed electrodes for the detection of antibiotics, as well as for the quantitative determination of bacteria and evaluation of their susceptibility to antibiotics. The attractiveness of the use of SPEs is due to their versatility, selectivity and sensitivity that are achieved through the numerous strategies of electrode modification with nanomaterials, chemical ligands, phages, antibodies, peptides, aptamers or bacteria as demonstrated by the presented results. The sensing concepts developed so far rely on various electrochemical detection techniques, voltammetry, amperometry and EIS being preferred. Most of the current efforts for developing new sensing devices/platforms are towards reaching high sensitivity and low detection limits. Nonetheless, achieving this and maintaining in the same time the simplicity in the sensor design, at a low price remains a permanent challenge. Innovation in screen-printing materials and technology can provide the solution to this by integration of some of time-consuming electrode modification steps into the mass production technology. Another need that remains to be addressed is for compact and in-field devices that are suitable for fast screening of biological and environmental samples. 

While from the point of view of sensitivity, rapidity and portability, SPE-based biosensors outperform many traditional antibiotics detection methods, their detailed validation was neglected in most studies. This includes evaluation of sample matrix effect, sensor/method robustness characterization and comparison with current standard methods. 

Selection of novel aptamers for antibiotics and bacteria is intensely researched and it can be anticipated that applications combining screen-printed electrodes—with commercially proven potential and many available configurations—with aptamers, which recently emerged in commercial tests for mycotoxin detection, can represent a successful practical concept. In the development of such biosensors, the possibilities for functionalizing the screen-printed electrodes in a manner compatible with mass production will play a central role.

Although many assays with electrochemical detection have been developed for the detection of bacteria, only a small part of these approaches can differentiate live versus dead cells and confirmation of such ability requires parallel analysis by classic culture growth. There are only a few more detailed works that studied the bacteria not only in pure cultures but also in mixed cultures or inoculated samples. As these reports found strong matrix effects and different performances in mixed compared to pure bacterial cultures, these challenges remain to be addressed in the future. 

The approaches towards parallel analysis of higher number of live bacteria of samples such as in [114] presented in this review can be extended to other bacteria using their specific phages as biorecognition elements. Moreover, further applications of this method for the detection of antibacterial effect or for screening antibiotic resistance are still to come. As the trend towards integrated microfluidic setups including bacterial capture, lysis and detection parts will continue, screen-printing as a versatile fabrication method will remain useful in their production.

Based on the different approaches emphasized in this review, bringing advantages that could be better exploited by combining the different ideas, and considering that the potential of screen-printed electrodes is increasingly exploited in sensing, it is clear that more applications will emerge to advance towards commercial analytical tools.

## Figures and Tables

**Table 1:** Examples of (bio)sensors based on screen-printed electrodes for detection of antibiotics.

Working Electrode ^1^	Antibiotic	Matrix	Linear Range (LR)/Detection Limit (DL)	Reference
*Amperometry*				
SPCE/AuNP/tyrosinase	Sulfamethoxazole	Buffer	LR: 20–200 µM DL: 22.6 µM	[33]
Dual SPCE/Protein G	Sulfapyridine	Milk	LR: 1.92–454 nM DL: 0.39 nM	[34]
	Tetracycline		LR: 6.4–385 nM DL: 1.93 nM	

Graphene/polyaniline modified screen-printed electrode coupled with UPLC	Sulfaguanidine	Buffer	LR: 0.01–10 µg/L DL: 1.162 µg/L	[35]
	Sulfadiazine		LR: 0.01–10 µg/L DL: 1.601 µg/L	
	Sulfamerazine		LR: 0.01–10 µg/L DL: 2.900 µg/L	
	Sulfamonomethoxine		LR: 0.01–10 µg/L DL: 2.467 µg/L	
	Sulfadoxine		LR: 0.01–10 µg/L DL: 2.995 µg/L	
	Sulfamethoxazole		LR: 0.01–10 µg/L DL: 2.513 µg/L	
	Sulfisoxazole		LR: 0.01–10 µg/L DL: 3.287 µg/L	
	Sulfadimethoxine		LR: 0.01–10 µg/L DL: 6.127 µg/L	
Affinity Penicillin-Binding Protein Magnetosensor	Penicillin	Milk	LR: 2.3–57.3 ng mL^−1^ DL: 0.93 ng mL^−1^	[36]
*Differential pulse voltammetry*				
SPCE/MWCNT/PBNC	Sulfamethoxazole	Urine	LR: 0.1–10.0 µmol L^−1^ DL: 38 nmol L^−1^	[37]
	Trimethoprim		LR: 0.1–10.0 µmol L^−1^ DL: 60 nmol L^−1^	
Screen-printed graphite electrode/antibody	Erythromycin	Bovine muscle	LR: N.D. DL: 0.2 ng mL^−1^	[38]
	Tylosin		LR: N.D. DL: 2 ng mL^−1^	
SPCE	Gemifloxacin	Buffer	LR: 0.5–10.0 µM DL: 0.15 µM	[39]
Aptamer	Ciprofloxacin	Milk	LR: 0.8–400 nM DL: 351 pM	[40]
		Serum	LR: 0.8–400 nM DL: 336 pM	
		Water	LR: 0.8–400 nM DL: 261 pM	
Au/aptamer/cDNA strands, arch-shaped/exonuclease I	Streptomycin	Buffer	LR: 30–1500 nM DL: 11.4 nM 14.3 nM (milk) 15.1 nM (serum)	[41]
aptamer/cDNA strands (M-shaped)	Tetracycline	Buffer	LR: 1.5 nM–3.5 µM DL: 0.45 nM	[42]
Graphene-SPCE	Tetracycline	Milk, Serum	LR: 10–120 µM DL: 3 µM	[43]
SPCE/aptamer	Tetracycline	Buffer	LR: 1 µM–5 mM DL: 0.6 nM	[44]
*Electrochemiluminescence*				
Ratiometric ECL aptasensor	Chloramphenicol	Buffer	LR: 0.1–120 nM DL: 0.03 nM	[79]
“Dual-potential” ECL aptasensor	Chloramphenicol	Buffer	LR: 0.2–150 nM DL: 0.07 nM	[76]
“Dual-potential” ECL aptasensor	Malachite Green	Buffer	LR: 0.1–100 nM DL: 0.03 nM	
*Electrochemical Impedance Spectroscopy*				
DNA aptamer	Penicillin	Buffer	LR: 0.4–1000 µg/L DL: 0.17 µg/L	[49]
DNA aptamer	Kanamycin	Milk	LR: 1.2–75 ng mL^−1^ DL: 0.11 ng mL^−1^	[50]
*Linear sweep voltammetry*				
thiolated aptamer /SPCE/AuNPs/magnetic double-charged diazoniabicyclo [2.2.2] octane dichloride silica hybrid	Epirubicin	Buffer	LR: 0.07–1.0 µM 3.0–21.0 µM DL: 0.04 µM	[45]
*Potentiometric titration*				
Calixarene/carbon nanotubes screen-printed sensors	Gentamicin Sulfate	Water	LR: 10^−7^–10^−2^ µM 75 nM	[48]
*Square wave voltammetry*				
bssDNA aptamer/SA-SPAuE	Tetracycline	Buffer	LR: 10 nM–10 µM DL: 10 nM	[46]
SPCE/AuNPs/cysteine SAM	Tetracycline	Urine	LR: 4–800 µM 0.42 µM	[47]
		Serum	LR: 4–700 µM DL: 0.54 µM	
		Milk	LR: 4–700 µM DL: 0.52 µM	
	Cefixime	Urine	LR: 2–700 µM DL: 0.32 µM	
		Serum	LR: 2–500 µM DL: 0.38 µM	
		Milk	LR: 2–500 µM DL: 0.35 µM	

^1^ SPCE: screen-printed carbon electrode; AuNP: gold nanoparticles; SPAuE: screen-printed gold electrode; SAM: self-assembled monolayer; ECL: electrochemiluminescence; MWCNT/PBNC: multi-walled carbon nanotubes decorated with Prussian Blue nanocubes; cDNA: complementary DNA; bssDNA: biotinylated single strand DNA; SA: streptavidin; UPLC: ultra-performance liquid chromatography.

**Table 2:** Examples of biosensors based on screen-printed electrodes for the detection of bacteria and evaluation of antibiotic resistance.

Bacteria/Sample	Sensor Configuration ^1^	Analytical Performance	Reference
Electrochemical Impedance Spectroscopy			
*S. typhimurium*; Cell cultures	SPCE modified with Au NP; aptamer	DL: 600 cfu mL^−1^; 13.8% increase in R_CT_ for 1 × 10^5^ cfu mL^−1^ heat-killed bacteria, 100% R_CT_ increase in with live *bacteria*	[78]
*Staphylococcus arlettae*; Spiked water and apple juice	Graphene electrode; bacteriophage	DL: 2 cfu Range: 2.0–2.0 × 10^6^ cfu; Response time: 2 min Stability: 3 months	[99]
*E. coli*; Cell cultures	SPCE; T4 phage	DL: 10^4^ cfu mL^−1^, onset of lysis observed after 20 min	[110]
*E. coli* K12; milk	phage-functionalized screen-printed carbon microarrays; T4 phage-magnetic beads;	DL: 10^3^ cfu mL^−1^	[111]
*bla_NDM_* gene	SPCE; peptide nucleic acid,	DL: 200 nM	[57]
*bla_NDM_* gene	SPAuE; peptide nucleic acid	DL: 10 nM (synthetic targets), 100 pM (PCR products)	[112]
*E. coli* and methicillin-resistant *S. aureus;* cell culture	Interdigitated electrodes; antibody	Analysis time: <90 min; AST, 6 antibiotics tested; results compared with bacteria viability and conventional antibiogram assay	[113]
Amperometry			
*E. coli* K-12, MG1655; cell culture	SPCE; activity of β-d-galactosidase in filtered cell lysate	DL: 1 cfu/100 mL for an incubation time of 8 h.	[114]
Differential Pulse Voltammetry			
*E. coli*; Drinking water, apple juice, and skim milk	Thin film Pt electrode; engineered T7 phage; release of β-galactosidase	10^5^ cfu mL^−1^ for 3 h interaction; 10^2^ cfu mL^−1^ after 7 h	[115]
Square Wave Voltammetry			
*E. coli* and *Enterococcus* spp.; pure cultures, water alfalfa sprouts, inoculated with *E. coli* and *E. faecium*	Screen-printed stencil electrodes on transparent film; release of β-galactosidase and β-glucuronidase (*E. coli*) and β-glucuronidase (Enterococcus)	DL: 10 cfu mL^−1^ *E. coli* after 4 h pre-culturing and 1 cfu mL^−1^ Enterococcus after 8 h culturing; DL: 2.3 × 10^2^ cfu g^−1^ (*E. coli*) and 3.1 × 10^1^ cfu g^−1^ *E. faecium* after 4 h and 12 h of pre-enrichment	[116]
*E. coli*; cell cultures	SPCE modified with didodecyldimethylammonium bromide (DDAB);	Test time: 2–5 h; resistance to cefepime, ampicillin, amikacin, and erythromycin	[117]
Chronocoulometry			
*E. coli* JM105; cell culture	Screen-printed carbon electrode arrays modified with poly-l-lysine or chitosan;	IC_50_ chloramphenicol: 2.0 ± 0.2 mM; 17 antibiotics tested; 20 min test time; measurement of bacterial respiratory activity	[118]

^1^ SPCE: screen-printed carbon electrodes; DL: detection limit; PNP: p-nitrophenol; SPAuE: screen-printed gold electrode.
